# L-Arginine-induced acute pancreatitis and its associated lung injury in rats: Down-regulation of TLR-4/MAPK-p38/JNK signaling pathway via *Ginkgo biloba* extract EGb 761 

**DOI:** 10.22038/IJBMS.2024.76162.16480

**Published:** 2024

**Authors:** Rasha Ezzat Mostafa, Sahar Samir Abdelrahmen, Dalia Osama Saleh

**Affiliations:** 1 Pharmacology Department, Medical Research and Clinical Studies Institute, National Research Centre (ID: 60014618), Cairo, Egypt; 2 Pathology Department, Faculty of Veterinary Medicine, Cairo University, Giza, Egypt

**Keywords:** Acute pancreatitis, Ginkgo biloba, Inflammation, L-Arginine, Lung injury, Rats

## Abstract

**Objective(s):**

Acute pancreatitis (AP) is an abrupt inflammatory condition characterized by a storm of inflammatory cytokines leading to high morbidity and mortality. The current study aimed to examine the efficacy of *Ginkgo biloba* extract EGb 761 (GBE) in the treatment of L-arginine-induced AP and its associated lung injury.

**Materials and Methods:**

Forty rats were randomly assigned into four groups. The normal group received only saline intraperitoneally while the other groups received two intraperitoneal L-arginine injections (250 mg/100 g b.wt) separated by a 1-hour interval to provoke AP. GBE (200 and 400 mg/kg/day, PO) was administered for 2 weeks post-induction of pancreatitis. Sera and pancreatic tissues were isolated.

**Results:**

The outcome of the present study revealed that GBE ameliorated the elevated levels of serum amylase, lipase, and pancreatic inflammatory mediators viz., tumor necrosis factor-alpha (TNF-α), mitogen-activated protein kinase P38 (MAPK-P38), c-Jun N-terminal kinase 1 (JNK1), and nuclear factor-kappa B (NF-κB). Moreover, GBE restored the pancreatic gene expression of Toll-like receptor 4 (TLR4) and prostatic acid phosphatase-2 (PAP-2). Pancreatic and lung histopathological examinations confirmed the aforementioned parameters.

**Conclusion:**

GBE interfered with the mechanistic pathway of L-arginine-induced acute pancreatic and its associated lung injury. Due to its anti-inflammatory properties, GBE can be used as a novel therapeutic candidate for the treatment of AP through down-regulating TLR-4/MAPK-p38/JNK and MAPK- p38/NF-κB signaling cascades.

## Introduction

Acute pancreatitis (AP) is an abrupt pancreatic inflammation. It is regarded as one of the most prevalent acute illnesses with growing prevalence worldwide. In many nations, AP is regarded as a digestive disorder that is characterized by a pancreatic inflammatory response that mandates hospitalization (1). The two chief causes of AP are gallstone disease and excessive alcohol intake. Repeated AP assaults can occasionally cause pancreatic fibrosis and loss of function over time (2). Various researchers have characterized AP as a complex pathophysiological course that includes oxidative stress, disturbed microcirculation, elevated calcium, and significant discharge of pro-inflammatory cytokines despite limited elucidation of the AP pathogenesis. One of the key pathogenic causes is oxidative stress which shares in the initial stages of AP (3). The excessive generation of reactive oxygen species (ROS) that occurs in AP causes tissue necrosis, granulocyte migration, zymogen degranulation, and increased amylase and lipase activity, all of which contribute to inflammation and pancreatitis (4). Oxidative stress and pro-inflammatory cytokines thus reinforce one another to create a vicious cycle in AP (5).

As a result of the production of pro-inflammatory cytokines and chemokines that attract granulocytes, many pathophysiological responses, such as stimulation of digestive enzymes like lipase and amylase, take place in AP (6). Severe AP was linked to substantial acinar cell necrosis while mild AP was linked to considerable apoptotic acinar cell death (7). Additionally, inflammation and complications during AP are directly attributed to oxidative stress (3, 8). To start the inflammatory cascade, pro-inflammatory cytokines like tumor necrosis factor-alpha (TNF-α) and interleukin-1 beta (IL-1β) activate nuclear factor kappa B (NF-κB) and mitogen-activated protein kinases-p38 (MAPK-p38), which leads to overexpression of cytokines, chemokines, and other pro-inflammatory mediators (9). 

Moreover, numerous cytokines are released systemically after AP-induced inflammation. Multiple organ dysfunctions can develop from injury to distant organs (such as the lungs or kidneys) if the level of systemic cytokines is high enough. AP-related death may be brought on by multiple organ dysfunctions or systemic inflammatory responses (10). Acute respiratory distress syndrome (ARDS) is a significant part of the syndrome of multiple organ failure associated with AP (11). Severe acute lung injury results from lung inflammation and is characterized by increased alveolar fluids because of decreased alveolar-capillary membrane permeability, which alters lung functionality. ARDS is the main cause of AP-related deaths (12). 

On the other hand, over the past ten years, *G. biloba* L. (family: Ginkgoaceae) has gained unparalleled popularity as a medicinal herb due to recognition of the significant therapeutic properties that this plant has demonstrated (13). *G. biloba* extract EGb 761 (GBE) has been linked to favorable biological activities and pharmacologic effects, including anti-oxidation, anti-inflammation, anti-aging as well as anti-tumor (14). The extract of GBE possesses potent anti-oxidant and anti-inflammatory properties (15).

Meanwhile, inflammatory lung damage is a significant clinical issue that has not received much research in relation to AP, the objective of this study was to characterize pancreatic changes as well as pulmonary changes. Therefore, the present study sought to examine the potential protective effects of GBE against the L-arginine-induced model of AP, recognized as a representative of the severe form of AP. Furthermore, the possible underlying mechanisms of this protection were thoroughly investigated. 

## Materials and Methods

***Materials***

*Animals*

Forty male Wistar rats (180–200 g) were purchased from the National Research Centre breeding unit, Cairo, Egypt. Routine husbandry has been followed for all animals. 

*Ethics*

The experiment was performed in accordance with the National Research Centre’s Medical Research Ethics Committee (MREC) ethical guidelines for regular experimental animal studies and the committee approved the experimental protocol (Ethical Approval no. 72311122022) which complied with the instructions provided by the Guide for Care and Use of Laboratory Animals (US - NIH Publication No. 85-23, revised 1996) and the ARRIVE guidelines. The experiment was also carried out in accordance with the U.K. Animals (Scientific Procedures) Act, 1986 and associated guidelines, EU Directive 2010/63/EU for animal experiments.

*Drugs and chemicals*

L-arginine (L-arginine powder, product No. 11009, Sigma-Aldrich^®^, St. Louis, MO, USA) and *G. biloba* EGb 761 (GBE: *G. biloba*®, 120 mg capsules; Holland & Barrett, UK) were used in the current study. 

*Experimental design and treatment protocol*

Forty male rats were randomly divided into 4 groups (n=10). Group I (normal group) received only saline intraperitoneally. Groups II-IV received two intraperitoneal L-arginine injections (250 mg/100 g body weight) separated by a 1-hour interval between injections to induce AP (16). Group II (Control group) received saline for 2 weeks post-induction of AP. Groups III and IV received *G. biloba *EGb 761 (GBE; 200 and 400 mg/kg/day, PO) (17) for 2 weeks post induction of AP. Twenty four hours after the end of the experiment, animals were sacrificed via decapitation under thiopental sodium anesthesia (20 mg/kg; IP) (18). 

***Methods***

*Serum biochemical analysis*

Blood was withdrawn under anesthesia from the retro-orbital plexus. Blood samples were centrifuged to separate the serum. Sera were then preserved (-20 °C) till the analysis of pancreatic alpha-amylase (AMY2A: Cusabio, USA; Catalog No. CSB-EL001689RA), pancreatic lipase (PNLIP: Abbexa, UK; Catalog No. abx155784) and C-reactive protein (CRP: MyBioSource, USA; Catalog No. MBS824463) using commercially available rat ELISA kits as per the instructions of the manufacturer.

*Tissue biochemical analysis*

Rats were sacrificed under anesthesia and pancreas and lung tissues were extracted from all groups. One part of the pancreas was homogenized and preserved at -80 °C for further tissue analysis. Lungs as well as the other part of the pancreas were kept in formalin for histopathological and immunohistochemical studies.

Protein content in the tissue was determined according to the literature (19). Mitogen-activated protein kinase-P38 (MAPK-P38: MyBioSource, USA; Catalog No. MBS720509), C-Jun N-terminal kinases 1 (JNK1: MyBioSource, USA; Catalog No. MBS2508439), Nuclear factor kappa light chain enhancer of activated B cells (NF-κB: MyBioSource, USA; Catalog No. MBS453975), and Tumor necrosis factor-alpha (TNF-α, Abbexa, UK; Catalog No. abx050220) were measured in pancreatic tissues as per the instructions of the manufacturer.

*Real-time polymerase chain reaction (PCR) quantification of Toll-like receptor 4 (TLR 4) and pancreatitis associated protein-2 (PAP-2)*

Pancreatic tissues from all groups were homogenized in ice and used for the extraction of total RNA and quantified by a Beckman dual spectrophotometer. Superscript IV One-Step RT-PCR kit (Thermo Fisher Scientific, Waltham, MA) was used for reverse transcription of the extracted RNA and then followed by PCR. The values were expressed in Cycle threshold (Ct) for the target and housekeeping genes. Normalization for variation in the expression of the target gene was performed relative to the mean critical threshold (CT) expression value of the housekeeping gene, GAPDH. The relative quantitation (RQ) of each target gene is quantified via the ΔΔCt method. The primer sequences used for the RT-PCR are shown in Table 1.

*Histopathological examination of lung and pancreatic tissues*

Lung and pancreatic sections from all groups were dissected and preserved in a 10% formalin solution. The tissues were cut into 5 μm thick sections, stained using H&E stain, and inspected using a binocular Olympus CX31 microscope. 

*Immunohistochemical analysis of lung tissues*

Interleukin 1β (IL-1β) and myeloperoxidase (MPO) were assessed, lung sections fixed in formalin were de-waxed and dehydrated in ethanol and incubated in H_2_O_2_ (3%). The immune reactivity was visualized by using diaminobenzidine (DAB; Sigma Chemical Co., USA). 

***Statistical analysis***

Data were presented as mean ± SEM. Statistical analysis was carried out using one-way ANOVA, followed by the Tukey-Kramer *post hoc* test. *P*<0.05 was assumed to signify statistical significance. GraphPad Prism^® ^software (version 9 for Windows, San Diego, California, USA) was used to carry out the statistical tests. 

## Results

***Effects of G. biloba EGb 761 on pancreatic enzyme activities and C-reactive protein levels in rats’ serum***

The intraperitoneal injections of L-arginine (250 mg/100 g b.wt) separated by a 1-hour interval resulted in a substantial rise of serum α-amylase and lipase activities to 426% and 459%, respectively versus normal rats. Moreover, serum levels of CRP were elevated post-L-arginine injections to 783% as compared to normal rats.

**Figure 1:**
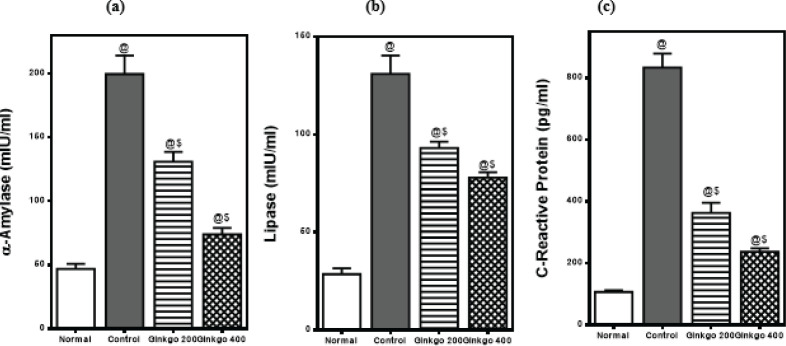
Effects of *Ginkgo biloba* EGb 761 on serum (a) α-amylase activity, (b) Lipase activity, and (c) CRP level in L-arginine-induced pancreatitis in rats’ serum

Treatment with GBE (200 mg/kg) reduced the elevated α-amylase and lipase activities to 66% and 71% and CRP level to 44% while GBE (400 mg/kg) reduced the elevated α-amylase and lipase activities to 47% and 56% and CRP level to 28% as compared to control rats (Figure 1).

***Effects of G. biloba EGb 761 on relative PAP-2 qRT-PCR mRNA gene expression in rats’ pancreatic tissues***

**Figure 2:**
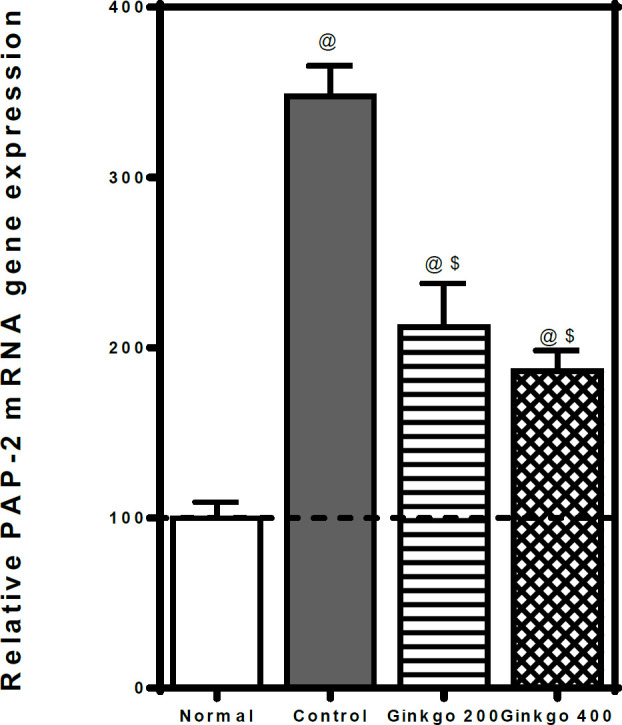
Effects of *Ginkgo biloba* EGb 761 on pancreatic relative PAP-2 qRT-PCR mRNA gene in L-arginine-induced pancreatitis in rats

The pancreatic PAP-2 mRNA gene expression was significantly elevated in L–arginine–control rats to ≈ 3.5 folds relative to normal rats. Whereas GBE treatment (200 & 400 mg/kg) dropped the elevated PAP-2 mRNA gene expression to ≈ 0.6 & 0.5 folds respectively as compared to control rats (Figure 2).

***Effects of G. biloba EGb 761 on relative TLR4 qRT-PCR mRNA gene expression in rats’ pancreatic tissues***

**Figure 3:**
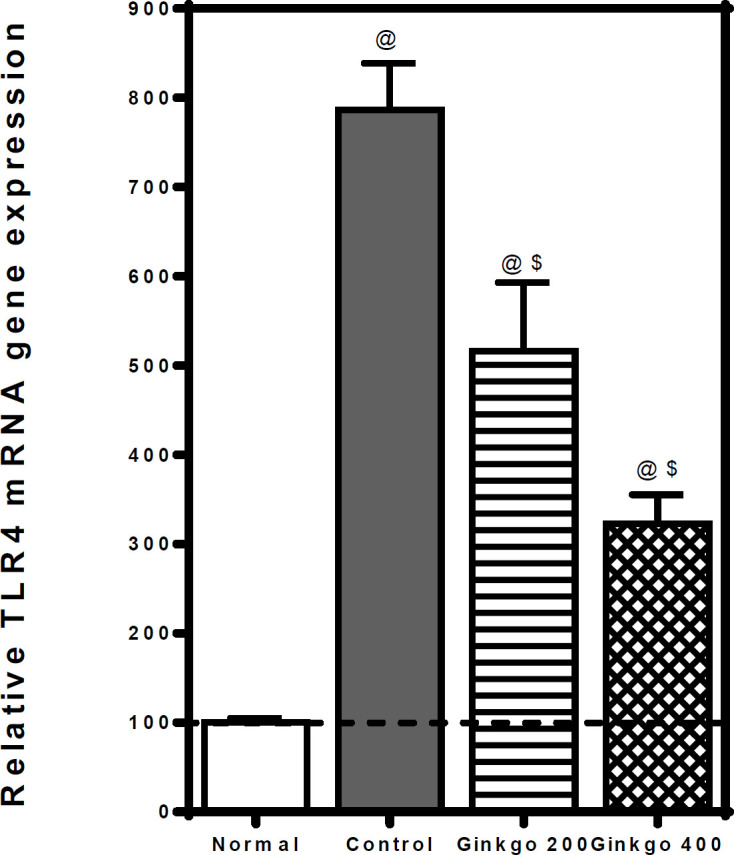
Effects of *Ginkgo biloba *EGb 761 on pancreatic relative TLR4 qRT-PCR mRNA gene in L-arginine-induced pancreatitis in rats

The pancreatic TLR4 mRNA gene expression was significantly elevated in L–arginine–control rats to ≈ 7.9 folds relative to normal rats. GBE treatment (200 & 400 mg/kg) dropped the elevated TLR4 mRNA gene expression to ≈ 0.7 & 0.4 folds, respectively as compared to control rats (Figure 3).

***Effects of G. biloba EGb 761 on MAPK-p38 / JNK/ NF-κB/ TNF-α signaling pathway in rats’ pancreatic tissues***

The intraperitoneal injections of L-arginine resulted in a significant rise of pancreatic tissue contents of MAPK-p38, JNK, NF-κB, and TNF-α to 351%, 521%, 406%, and 427%, respectively as compared to normal rats. 

**Figure 4:**
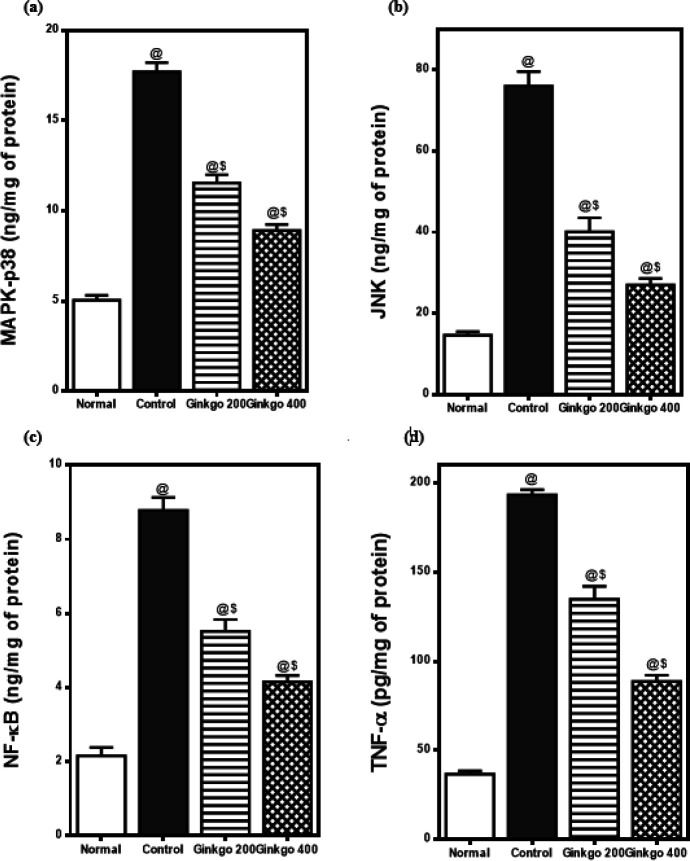
Effects of *Ginkgo biloba* on pancreatic (a) MAPK-p38, (b) JNK, (c) NF-κB, and (d) TNF-α levels in L-arginine-induced pancreatitis in rats.

*G. biloba* EGb 761 treatment (200 mg/kg) dropped the elevated pancreatic tissue levels of MAPK-p38, JNK, NF-κB, and TNF-α to 65%, 53%, 63%, and 70%, respectively. On the other hand, GBE treatment (400 mg/kg) decreased the raised pancreatic tissue levels of MAPK-p38, JNK, NF-κB, and TNF-α to 50%, 36%, 47%, and 46%, respectively as compared to control rats (Figure 4).

***Effects of G. biloba EGb 761 on histopathological examination of lung and pancreatic tissues***

**Figure 5:**
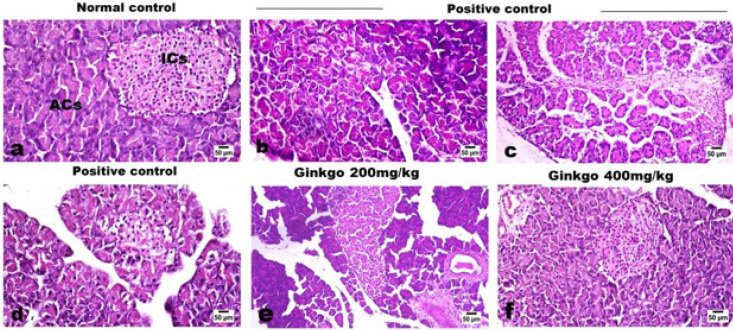
**.** Effects of *Ginkgo biloba* EGb 761 on histopathological alterations of pancreas tissues in L-arginine-treated rats (Stain: H&E)

*G. biloba* EGb 761 treatment strongly amended the histological alterations induced by L-arginine. Microscopic examination of pancreatic tissues of normal control rats revealed the typical histological assembly of the endocrine pancreatic islets of Langerhans and the pancreatic exocrine acinar cells (Figure 5a). While examination of the pancreatic tissue of control-positive rats revealed vacuolar degeneration, scattered necrosis, and nuclear pyknosis of the acinar cells, with loss of zymogen granules and few apoptotic cells (Figure 5b). Mild to moderate degree of mononuclear inflammatory cells infiltrating the interstitial tissue was noticed with mild fibrous proliferation (Figure 5c). Interacinar and mild interlobular edema were evident. The islet cells in the vicinity showed swelling, vacuolar degeneration, necrosis, and loss of the islet’s cells (Figure 5d). Regarding the groups that were treated with GBE 200 (Figure 5e) and 400 mg/kg (Figure 5f), the examination of their pancreatic tissues revealed a dose-related restoration of both exocrine and endocrine pancreatic parts with marked retraction of the inflammatory reaction.

**Figure 6:**
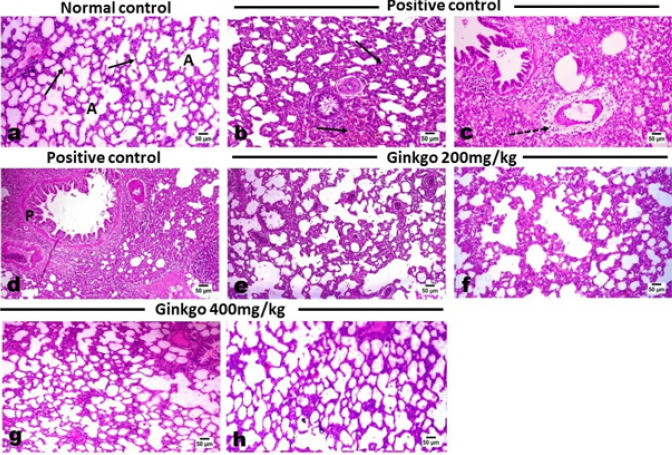
Effects of *Ginkgo biloba* EGb 761 on histopathological alterations of lung tissues in L-arginine-treated rats (Stain: H&E)

Microscopic inspection of the lungs of control rats showed a typical histological structure of the inter-alveolar septa and lung alveoli (Figure 6a). While examination of various sections of the lung of control-positive rats revealed noticeable thickening of the inter-alveolar septa (Figure 6b) with apparent areas of emphysema. The later thickening was a result of hyperplastic alveolar epithelium and an elevated number of lymphocytes, macrophages, and fibroblasts as well as pneumocytes type II**.**

Most of the blood vessels showed thickening of their walls with perivascular edema and inflammatory cell infiltration (Figure 6c) in a picture of perivasculitis. Marked hyperplasia of the peribronchial lymphoreticular tissue was evident (Figure 6d). Examination of the lungs of control-positive rats that were treated with GBE at doses of 200 and 400 mg/kg and MD revealed marked dose-related relief of the inter-alveolar walls’ thickening. At the low dose, there was a mild to moderate degree of septal thickening with retraction of the inflammatory reaction (Figures 6e and f). While a more obvious decrease in the septal thickening and inflammatory infiltrates was noticed in the high-dose treated group (Figures 6g and h). 

***Effects of G. biloba EGb 761 on immunohistochemical analysis of IL-1β and MPO in lung tissues***

**Figure 7:**
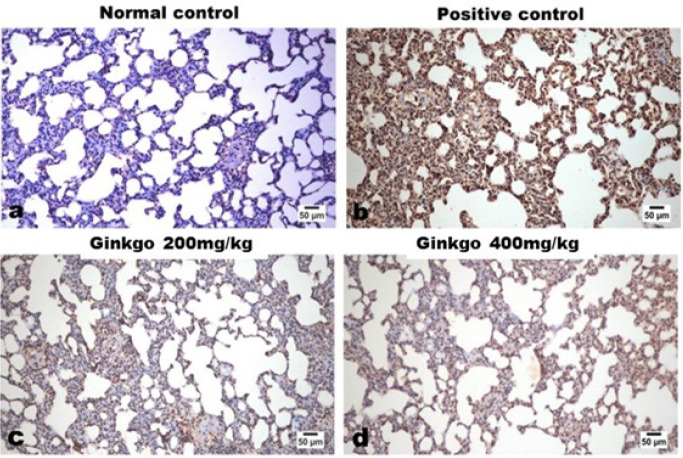
Effects of *Ginkgo biloba* EGb 761 on immunohistochemical lung tissue expression of MPO in rats

**Figure 8:**
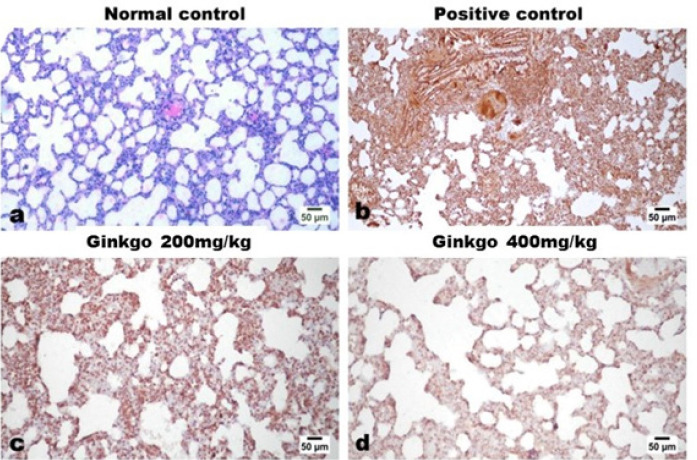
Effects of *Ginkgo biloba *EGb 761 on immunohistochemical lung tissue expression of IL-1β in rats

Examination of immune-stained lung sections of control rats revealed negative expression of MPO (Figure 7) and IL-1β (Figure 8). While examination of the lung tissue of control-positive rats revealed a uniformly strong and diffuse expression of both markers. A marked dose-related decreased expression of both markers was noticed in lung tissues of GBE-treated groups as presented by the figures and quantified by the image analysis software (Table 2).

**Table 1:** Primer sequences used for RT-PCR quantification of Toll-like receptor-4 (TLR-4) and pancreatitis associated protein-2 (PAP-2)

TLR-4	**Forward gene**	**5′- TGAAGTATATCCACTCGGCGGG -3′**
	Reverse gene	5′- TGGATGCACTACAACCAGACAG -3′
PAP-2	Forward gene	5′‐ CAAGGCCTGCCAGAGGAAGTT ‐3′
	Reverse gene	5′‐ ATGAGCCAAAAGGTCGGA ‐3′
GAPDH housekeeping	Forward gene	5'-TGTGTCCGTCGTGGATCTGA-3'
	Reverse gene	5'-TTGATGTTGAAGTCGCAGGAG-3'.

**Table 2:** Effects of *Ginkgo biloba *EGb 761 on lung tissue MPO and IL-1β immunohistochemical staining in L-arginine-induced pancreatitis in rats

Parameter	MPO expression	IL-1β expression
Groups		
Normal	0.04±0.12	0.06±0.12
Control	5.28±1.02^@^	5.64±0.94^@^
Ginkgo 200	2.86±0.50^@$^	3.28±0.78^@$^
Ginkgo 400	1.98±0.36^@$^	2.06±0.55^@$^

Data is presented as mean ± SEM. Data is analyzed by non-parametric K independent samples Kruskal Wallis test then Dunn’s multiple comparison *post hoc* test, ^@^Significantly different vs normal group at *P*<0.05 and ^$^Significantly different vs L-arginine control group at *P*<0.05

MPO: Myeloperoxidase; EGb: Ginkgo biloba EGb 761

Data is presented as mean ± SEM (n=10). Data is analyzed by one-way ANOVA followed by Tukey-Kramer’s *post hoc* test, ^@ ^Significantly different vs normal group at* P*<0.05 and ^$ ^Significantly different vs L-arginine control group at *P*<0.05.

Data is presented as mean ± SEM (n=10). Data is analyzed by one-way ANOVA followed by Tukey-Kramer’s *post hoc* test, ^@^ Significantly different vs normal group at *P*<0.05 and ^$^ Significantly different vs L-arginine control group at *P*<0.05.

EGb: Ginkgo biloba EGb 761

Data is presented as mean ± SEM (n=10). Data is analyzed by one-way ANOVA followed by Tukey-Kramer’s *post hoc* test, ^@^ Significantly different vs normal group at *P*<0.05 and^ $^ Significantly different vs L-arginine control group at *P*<0.05.

Data is presented as mean ± SEM (n=10). Data is analyzed by one-way ANOVA followed by Tukey-Kramer’s *post hoc* test, ^@ ^Significantly different vs normal group at *P*<0.05 and^ $ ^Significantly different vs L-arginine control group at *P*<0.05.

**Figure 9:**
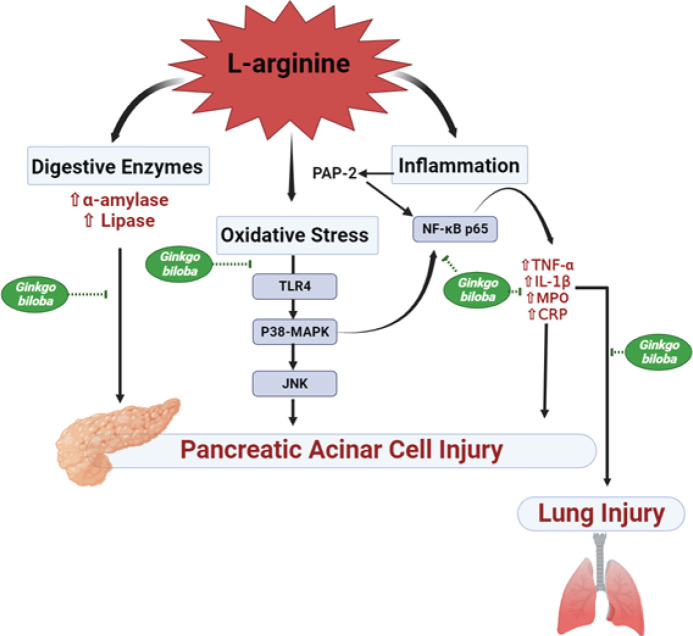
Proposed mechanism of the protective role of *Ginkgo biloba *against L-arginine-induced AP and its associated lung injury via hindering of TLR-4/MAPK-p38/JNK and p38MAPK/NF-κB signaling pathway

## Discussion

AP is an acute non-bacterial condition that is regarded as one of the most prevalent acute illnesses with rising prevalence worldwide (20). The objective of the current experimental study was to assess GBE potential as a treatment for AP caused by L-arginine. L-arginine administration triggered pancreatic damage in the current investigation, which was demonstrated by the striking rise in serum lipase and amylase activities which are the most important diagnostic indicators for AP (16). The aforementioned finding was consistent with other investigations (21-23). The secretion of digestive enzymes in the pancreatic acinar cells, which results in self-digestion of the pancreas, is the first effect that initiates AP (6). 

In the AP cascade, which mediates inflammatory cell adhesion and long-lasting tissue injury, ROS are also involved (24). By investigating the inflammatory mediators, the defenses of GBE against AP are revealed. GBE treatment significantly reduced the severity of this pancreas injury evidenced by a reduction in the digestive enzymes including serum lipase and amylase. This suggests that GBE may be able to obstruct one or more signaling pathways linked to AP’s development. 

The cytokines, which are primarily generated by active leukocytes, are crucial parts of the inflammatory cascade. TNF-α is considered the most important member of the inflammatory cytokine family because it initiates and propagates nearly all of the symptoms of the systemic inflammatory response syndrome (25). According to the current study, the AP group had significantly higher pancreatic TNF-α levels. L-arginine triggers the production of pro-inflammatory cytokines. This is a crucial factor for stimulating a series of pathological progressions in AP that enhance inflammatory pathways and result in tissue damage (26). Similarly, serum CRP has been also identified as a key contributor to AP and typically follows the release of TNF-α among other pro-inflammatory cytokines (27). According to Chen, an elevated level of CRP can predict the severity of AP and the likelihood of comorbidities (28). Contrarily, pretreatment with GBE reduced the rise of TNF-α and CRP levels in the current study. These results were in agreement with prior investigations which demonstrated the anti-inflammatory properties of GBE (13, 15).

Moreover, L-arginine induced AP via elevation of the inflammatory TLR-4 gene expression. These results are coherent with preceding studies conducted on AP in experimental rat and mouse models (29). Inflammation in pancreatic islets is connected to TLR4. In pancreatic beta-cells and macrophages, the TLR4/NF-κB signaling pathway (30) is a potentially vital mediator in the progression of this form of pancreatic damage (31). Additionally, bone marrow or adipose tissue-derived M2 macrophages can penetrate pancreatic-islet tissue and develop into M1 macrophages. The level of inflammatory cytokines increases in activated M1 macrophages, aggravating inflammation and worsening cell dysfunction in pancreatic islets (32). In the current study, GBE treatment significantly reduced the enhanced TLR4 mRNA gene expression dose-dependently, in comparison to AP rats.

It is also proposed that stimulation of MAPKs is a key incident in the initiation and onset of AP and occurs early in the disease (33). MAPK-p38 activation can boost the NF-κB activity (34). In order to further stimulate the release of inflammatory cytokines, TNF-α may furthermore activate NF-κB (35). It is noteworthy that the majority of stressors and pro-inflammatory stimuli activate MAPK-p38 and JNK (36). Conversely, NF-κB and MAPK pathways are the two primary signaling pathways in AP that control the transcription and production of inflammatory mediators. When MAPK pathways are engaged, they coordinate the initiation of gene transcription, activating cellular processes including proliferation, cell differentiation, and inflammation which are controlled by the secretion of additional hormones and growth factors (37). Interestingly, inhibitors of MAPK have drawn a lot of attention in recent years, mainly because they have been identified as pivotal regulators of inflammatory disorders including AP. It has been shown that the progression of AP is aided by the activation of MAPK signaling axes as an early incident in AP (38). Moreover, it has been shown that selective JNK inhibition causes the amendment of AP (39). 

The present investigation demonstrated that pretreatment with GBE, compared to the AP group, could significantly inhibit the MAPK-p38/NF-κB pathway as well as TLR-4/MAPK-p38/JNK-1, limit the induction of inflammatory mediators, and decrease the pancreatic damage. This discovery was supported by several studies, which discovered that GBE has the power to prevent cellular inflammation and the subsequent activation of MAPK-p38 (40, 41). Furthermore, it has been reported that GBE is a prospective protecting agent against acute lung injury via inhibition of the JNK-1 and Akt-dependent NF-κB activation pathways (42). Li *et al.* (2020) have attributed the anti-inflammatory immunomodulation of GBE to ginkgolides, one of the significant medicinal constituents in *G. biloba* (41). Therefore, down-regulation of pro-inflammatory cytokines and the suppression of the cross-talk between oxidative stress and inflammation which increases the inflammatory burden that contributes to cellular injury are the causes of GBE modulation of the aforementioned inflammatory mediators as illustrated in Figure 9.

The fact that L-arginine increased PAP2 gene expression in comparison to the control group is another interesting discovery in the current investigation. One of the three homologous PAP isoforms, PAP2, is significantly increased during AP and is a potential moderator of early inflammation (43). Notably, PAP2 has been shown to mediate NF-κB translocation into the nucleus (44). It is well known that during AP, NF-κB activation is adept at producing a variety of cytokines, including Il-1β, TNF-α as well as CRP, potent mediators of inflammation that play a significant role in either starting AP or worsening its course (45). However, GBE pretreatment reduced the expression of the pancreatic PAP2 gene (46), which may be connected to its potent anti-inflammatory properties (47). 

On the dark side, AP comes with systemic inflammatory responses leading to multiple organ failure. A major component of the condition of multiple organ failure related to AP is acute respiratory distress syndrome (10). An inflammatory response is crucial and significantly affects how pulmonary injury in AP develops. Lung damage is significantly influenced by pro-inflammatory elements. Pro-inflammatory cytokines, which primarily affect human lung microvascular endothelial cells, are thought to cause and perpetuate lung injury (48). The blood-air barrier is destroyed during AP by the lung’s buildup of inflammatory cells and inflammatory mediators. Numerous investigations demonstrated that the neutrophil buildup in the lungs increased the secretion of pro-inflammatory cytokines, such as IL-1β which activated several intracellular signaling pathways such as NF-κB and MAPK, causing the release of inflammatory intermediates, which exacerbated the damage (49). Elevated lung MPO activity and inflammatory cells’ infiltrates have both been noted in experimental AP as signs of lung injury brought on by AP (50). To our knowledge, few studies have examined lung injury associated with AP in animal models. Lung MPO and Il-1β immunostaining as well as alterations in the lung morphology associated with L-arginine-pancreatic injury have been observed in the current study which has been substantially restored post-GBE treatment, dose-dependently. 

The outcomes of the current study are supported by the histological alterations that took place. The pancreatic acini and islets of Langerhans in the control and GBE groups showed no pathological alterations. The L-arginine group, on the other hand, displayed a variety of histological alterations, including atrophic islets, acinar degeneration, crooked pancreatic ducts, and cellular infiltration. This is consistent with findings from former studies (51, 52) that supported the same outcomes.

## Conclusion

The current investigation proposed the pivotal role of inflammatory cytokines in the pathophysiology of AP associated with multiple organ dysfunction syndrome, the most important of which is lung injury. The present investigation confirmed the preventive effect of GBE against L-arginine-induced AP as well as the associated lung injury. Furthermore, the mechanism by which GBE exerted its anti-inflammatory effects occurs via down-regulation of TLR-4/MAPK-p38/JNK as well as MAPK-p38/NF-κB signaling axes, as summarized in Figure 9, thus leading to hindering related cytokines. The present study suggests GBE as a potential AP preventative or adjuvant therapeutic target. 

## Authors’ Contributions

All authors contributed equally to the study’s conception and design. All authors have read and approved the final version of the manuscript.

## Funding

The authors have no relevant financial or non-financial interests to disclose.

## Availability of Data and Material

All data will be available upon request.

## Conflicts of Interest

The authors have no conflicts of interest to declare that are relevant to the content of this article.
